# A cross-sectional survey of smoking and cessation support policies in a sample of homeless services in the United Kingdom

**DOI:** 10.1186/s12913-022-08038-7

**Published:** 2022-05-13

**Authors:** Sharon Cox, Jaimi Murray, Allison Ford, Lucy Holmes, Deborah Robson, Lynne Dawkins

**Affiliations:** 1 Department of Behavioural Science and Health, University College London, London, UK; 2 Spectrum Research Consortium, London, UK; 3 Centre for Addictive Behaviours Research, London South Bank University, London, UK; 4 Institute of Social Marketing and Health, University of Stirling, Stirling, UK; 5 Alcohol Change UKGroundswell, London, UK; 6 Institute of Psychiatry, Psychology and Neuroscience, Kings College London, London, UK

**Keywords:** Tobacco, Homelessness, Smoking cessation, Harm reduction, Smoking, Vaping, E-cigarette, Policies, Survey

## Abstract

**Background:**

Smoking is extremely common amongst adults experiencing homelessness. To date, there is no nationally representative data on how tobacco dependence is treated and if and how smoking cessation is supported across the homeless sector. The aim of this study was to document smoking and e-cigarette policies of UK homeless services and identify areas of good practice and where improvements could be made.

**Methods:**

A cross-sectional survey with homeless centre staff was conducted between June 2020-December 2020 totalling 99 homeless centres. Quotas were stratified based on population and service type across Scotland, Northern Ireland, Wales, and England. Interviews were conducted over the phone or online in a minority of cases. Survey questions were themed to assess, i) onsite smoking and e-cigarette (vaping) policies ii) screening and recording of smoking status, iii) cessation training and resources available to staff, iv) cessation support for service users.

**Results:**

92% accounted for smoking within their policies in some form (stand-alone policy (56%) or embedded within another health and safety policy (36%)). 84% allowed smoking in at least some (indoor and outdoor) areas. In areas where smoking was not allowed, vaping was also disallowed in 96% of cases. Staff smoking rates were 23% and 62% of centres reported staff smoked with service users. Just over half (52%) reported screening and recording smoking status and 58% made referrals to Stop Smoking Services (SSS), although established links with SSS were low (12%) and most centres did not provide staff training on supporting smoking cessation. Areas of good practice included regular offers of smoking cessation support embedded in routine health reviews or visits from SSS and offering tangible harm reduction support. Areas for improvement include staff training, staff smoking with service users and skipping routine screening questions around smoking.

**Conclusions:**

Smoking is accounted for across different policy types and restricted in some areas within most settings. Smoking cessation support is not routinely offered across the sector and there is little involvement with the SSS.

**Supplementary Information:**

The online version contains supplementary material available at 10.1186/s12913-022-08038-7.

## Background

Tobacco smoking is a leading cause of premature death and disease and is strongly associated with deprivation and health inequalities [1–3]. Homelessness and housing shortages are growing problems in the UK, leading to exacerbated poverty and poor health [4]. Smoking rates are exceptionally high amongst adults accessing homeless support services, with rates ranging between 57 and 82% [5], this is up to four times higher than the national UK average (14.1%; [6]). The harms caused by tobacco smoking are likely to be exacerbated in this group due to higher prevalence of chronic obstructive pulmonary disease (COPD), heart problems and respiratory viral illnesses [7, 8]. This may be linked to frequent engagement in risky smoking practices, i.e., puffing harder and longer, smoking unfiltered cigarettes, smoking discarded cigarettes and sharing cigarettes [5, 8], as well as concurrent use of illicit substances (e.g., heroin, crack) which also negatively impacts lung function [9]. Owing to these high prevalence rates and exacerbated risks, people who smoke who access homeless services represent a key group in need of smoking cessation interventions. Homeless support services may be a useful place to support smoking cessation as many professionals already have good relationships with their service users and there is an established foundation for offering support. To date, there is little published literature on how smoking and cessation is treated and managed across homeless services in the UK. Before recommendations on how to effectively support smoking cessation within this sector can be made, establishing a baseline of what is being offered to support smoking cessation is needed.

Owing to the complex nature of homelessness and needs of the people experiencing it, research shows that smoking takes low priority in the assessment of health needs [5, 10, 11]. In some incidences, smoking is viewed as beneficial by the people who smoke and the people who support them because it is perceived as stress relieving and providing comfort [11, 12]. Other erroneous perceptions also exist among professionals, such as the belief that people experiencing homelessness do not want to quit smoking or would not take up the offer of support [13]. Alongside these competing issues, social determinants of smoking are also a barrier to quitting and staying quit. For example, a high percentage of smoking peers, smoking in most social environments and common practices such as sharing cigarettes afford smoking a culturally importance practice [5, 8, 11, 12]. In our previous study by Dawkins et al., of 283 smokers accessing homeless services, 75% expressed a desire to quit smoking and quit attempts (albeit short-lived) were common [14]. However, respondents also reported that many of their attempts were unaided, meaning participants did not use evidence-based treatments such as licensed nicotine replacement therapies, e-cigarettes or behavioural support [15–17]. They also reported they had little contact with Stop Smoking Services (SSS), which are free to access and offer behavioural support and licensed stops smoking medication, a range of support and prescribed medications often provided free of charge for those on low incomes and in receipt of state benefits. E-cigarettes are also provided free of charge in many SSS in England (40% at the time of writing [18]). There is also low endorsement of traditional nicotine replacement therapies but higher endorsement for the use of e-cigarettes in future quit attempts [14]. Qualitative work has shown that e-cigarettes are viewed more positively by people considering quitting smoking while experiencing homelessness [12, 14] and are favoured over more medicalised approaches because they offer a less structured and less formal approach to continue using nicotine without the harms of smoking tobacco. For homeless services already offering established substance harm reduction interventions (e.g., safer injecting equipment, condoms, opioid substitution therapies), e-cigarettes may fit well with this model, as they likewise offer a less harmful way to use a substance while reducing associated risks [18]. The Royal College of Physicians (2021) state that e-cigarettes are an effective treatment for tobacco dependency and their use should be included and encouraged in all treatment pathways [19]. The National Institute of Health and Care Excellence in England recommend that nicotine containing e-cigarettes should be accessible to adults who want to quit smoking [20].

We recently conducted a cluster feasibility study to explore the uptake and use of e-cigarettes compared with usual care (i.e., information about the local SSS and help to quit leaflet) offered within four homeless services in England and Scotland [12, 21]. Our findings provide support that service users accessing the centres would take part in the study, uptake was good with 52% of eligible participants signing up to the study, and retention was also comparable to other studies with this population (e.g., [5]). Within our embedded process evaluation, it was highlighted by staff that the pre-study training they received from the research team helped them to understand the importance of smoking and cessation within this population and equipped them to offer either the e-cigarette intervention or the usual care information. Participants and the research team also noted that better centre policies and regulations around smoking and vaping could act as potential facilitators for transitioning away from smoking. Specifically, at one centre those in the e-cigarette intervention created a ‘vaping community’ and a vaping drop-in service was offered by staff. To date, there is no nationally representative evidence from the UK on how smoking cessation and vaping is supported by policies and practices in homelessness services. To address smoking rates and to reduce high rates of cancer, other diseases and respiratory illness within this population, a targeted approach to tobacco dependence is required.

Accordingly, the overall aim of this study was to ascertain the rates and type of smoking, e-cigarette and cessation policies in homeless services, specifically to document: i) whether onsite smoking and e-cigarette (vaping) policies exist, ii) the practices and rates around screening and recording of smoking status, iii) whether and what type of cessation training and resources are available to staff, iv) the types of cessation support offered to service users, and v) areas of good practice and areas for improvement.

## Method

### Design and setting

A cross sectional survey including structured and open-ended questions. The study was preregistered on the Open Science Framework (https://osf.io/ncmkj/). We collected data from ‘local’ charities, i.e. those which are service user facing (rather than, for example, head offices), and a smaller sample of national ‘strategic’ level charities. Only the former is reported here. We aimed to recruit 100 homeless services and the achieved sample was 99. The sample size was based on similar surveys in health or education settings (e.g., [22]).

Ethical approval was provided by London South Bank University. Data collection took place between May – December 2020, this was during the COVID-19 pandemic and a time of social restrictions across the UK. All participants provided informed consent and were treated in accordance with the Declaration of Helsinki (1964).

#### Sampling of charities

Homeless services were recruited from Scotland, Northern Ireland, Wales and England. Quotas were stratified based on service type (non-residential, short term and long-term residential services). Services were identified using Charity Choice (https://www.charitychoice.co.uk/), Charity Commission (https://www.gov.uk/government/organisations/charity-commission), Homeless Link (https://www.homeless.org.uk/), individual charity websites, social media and newsletter were also used to recruit charities to the study. Table 1 presents the characteristics of the participating services.

**Table 1:** Homeless service characteristics

	*n* = 99	
Location of service %	Scotland	8.1
	Northern Ireland	3
	Wales	5
	North of England	24.3
	South of England	19.2
	Midlands and East of England	24.2
	London	15.2
	Missing	1
Mean number of staff per service, including volunteers *(sd)*	29 (35.5)	
Mean number of service users visiting on an average day *(sd)*	49.2 (48.7)	
^a^Type of centre/s %	Supported housing	65.7
	Day centres	30.3
	Emergency night shelters	29.3
	Crash pad	9.1
^a^Type of services/support offered %	Employment	41.4
	Mental health	40.4
	Housing/accommodation	37.4
	Physical health	24.2
	Substance use	24.2
	Street outreach	13.1
	Winter shelter	4
	Food services	3
Estimated mean percentage of staff who smoke and vape *(sd)*	Smoke 22.6% (20.6%)	
	Vape 8.7% (12.4%)	

^a^More than one response can be selected, a definition of these types of services can be found in the supplementary material

### Procedure and materials

Eligible participants were senior staff of homeless services, specifically those with knowledge of smoking policies and onsite practices.

The online survey link was posted on various support organisation blogsites, newsletters, and the University’s social media channels. In addition, from searching the databases above participants were contacted by telephone and email and invited to participate.

Participants were invited to complete the survey by telephone or online via Qualtrics (over 90% responded by taking the telephone survey). All respondents were first sent an email with an embedded consent form. Surveys administered via telephone were audio recorded and responses transferred into Qualtrics. Responses were repeated to participants to confirm the correct information had been recorded. 

### Measures

The survey included open and closed/multiple choice questions (see Supplementary file), initially based on the Care Quality Commission Smokefree policies for mental health inpatient services [23] and adapted for homelessness services. The six survey categories were, a) smoking & vaping policies (e.g., do they exist, what do they include, are they adhered to); b) screening and recording of smoking status (e.g. timing and nature); c) smoking cessation training for staff; d) types of smoking cessation support for service users, and e) participants were offered a chance to tell us about good practice. The survey was further developed with public and stakeholder involvement.

### Analysis

Quantitative data was stored on SPSS Version 26 and presented descriptively. Data were cleaned to identify miscoded and missed responses.

Open question responses were imported into Microsoft Excel for inductive descriptive thematic analysis. Emerging themes of good practice and areas for improvement were identified and discussed by JM and AF until consensus was reached. A coding framework was prepared according to the study themes, and data were coded (JM) and checked (AF). The findings from the open-ended questions were then interpreted and discussed with the wider team. Direct participant responses have been selected to illustrate the findings (Table 2).

**Table 2:** Respondents’ examples of good practice and areas for improvement identified by the research team from open questions

	Examples of respondents’ comments
**Areas of good practice**	
Screening for smoking and proactive use of information	We always ask whether they smoke when they sign into the service. We also ask if they would like support to give up and if they say yes this will form part of their support plan. (P20)
	When they come for interview before they move in… we ask about how much they smoke and whether they would like to quit. We can refer at this point, but usually we wait until they move in. (P31)
	We ask all service users if they would like to change their smoking habits, but we are guided by the client. If they decline, we do review in future sessions… (P19)
	Smoking is covered in the physical health checklist in the needs assessment. It may also come up in the finance/budgeting section because the cost can be a barrier. (P42)
Relationships with stop smoking services	The relationship we have with the GP and the stop smoking clinic through them. As most residents are on medications, their smoking is brought up each time they have a medication review. In addition, each time the residents have a tuberculosis test (twice a year), smoking information sessions are held then also. It is spoken about often to residents so it is not something that slips under the radar. (P12)
	The most benefit we have found is around partnering with (name’s organisation). We also ran a Better Health at work campaign and we reduced staff smoking rates by 50% which I think benefits our clients because we are setting good examples. (P22)
	We found the cessation service visiting once a week to be helpful. It took a while to build it up, but most of what is taken up [is influenced by] their peers/other residents… They often don’t want to engage unless they hear someone else has a good experience. (P15)
	Having a healthy living week is good, and getting in the local cessation service works well. Especially when they bring the visual tools, it’s less about a lecture, it’s more interactive. It gives smoking and personal harm more context, that is measurable, rather than just being told that smoking is bad for you.(P43)
Vaping encouraged as an alternative to smoking	We had great success when we bought people vape kits… We saw a large number of people switch to vaping because we saw people supporting each other and helping each other, and that was more effective than sending someone to a group. (P5)
	We find harm reduction to be effective. Some service users have switched to the vape, and they eventually smoked less than they did previously. (P42)
	We did have some success with the local vape store. I think it was because we were able to provide something tangible for service users to try, rather than just running an information session. We provided the information, and then had vapes available for them to try which was quite good. (P40)
	The main service that were finding clients are interested in at the moment is going onto a vape through the NHS. As they get the vape and liquid for free, we have had more residents wanting to engage with the service and try to stop smoking than before. (P28)
**Areas for improvement**	
Lack of staff training on smoking and smoking cessation	We don’t have any formal training, but we do have leaflets around from our local GP cessation service and we tell staff where and how to signpost. (P53)
	If [smoking cessation] is something that we are to focus on, it would be beneficial if there was a greater push from higher. There would be benefits to creating targets, providing training and more tools around how to support smoking cessation. (P26)
	[We provide no training in smoking], we are more concerned with stopping them smoking crack. (P2)
Staff smoking with service users	Staff and residents share a common smoking area in the hostel so there are no rules around smoking in front of residents. Code of conduct means staff cannot give cigarettes or any other form of smoking material/s to residents. (P17)
	Staff smoke in the same place as service users and sometimes will be smoking at the same time. Smoking is sometimes used as a rapport building tool. (P29)
	We find that cigarette smoking helps de-escalate a situation if a service user is becoming distressed. So a staff member will have a cigarette with the client. (P4)
	It is not desirable to smoke in front of clients, however, if it is to get them to engage on common ground then it is acceptable in some circumstances. (P21)
Lack of screening for smoking or screening for risk assessment only	[Service users] complete a survey upon entering a service, but smoking is not covered in this. We only cover smoking if the service users identify it themselves. (P2)
	As part of their formal assessment when they first move in, there is a question about smoking, but this doesn’t always get asked. Sometimes it’s not appropriate ask service users about their smoking when they have really complex issues. (P26)
	[Smoking] is usually on their referral. We are a no smoking hostel so it would be discussed with them when they move in. There is no smoking at all on site. This was decided after a large fire at the centre… (P17)

## Results

Seven hundred and twenty-eight survey requests were directly made by the research team over the recruitment period, 29 declined, 600 did not respond and 99 participants from individual charities (i.e., separate organisations) consented and took part (no responses were gained via social media or newsletters). Table 1 provides the charity characteristics. Analysis of open question responses identified examples of good practice in relation to, 1) screening for smoking and proactive use of information, 2) relationships with SSS, and 3) vaping encouraged as an alternative to smoking. Responses also gave insight into areas for improvement such as, 1) lack of staff training on smoking and smoking cessation, 2) staff smoking with service users, and a 3) lack of screening for smoking or screening for risk assessment only (Table 2). Screening for smoking was highlighted as both good practice in some centres but also an area for improvement in others, suggesting a lack of consistency across the sector. 

### Onsite smoking and vaping policies

**Fig. 1:**
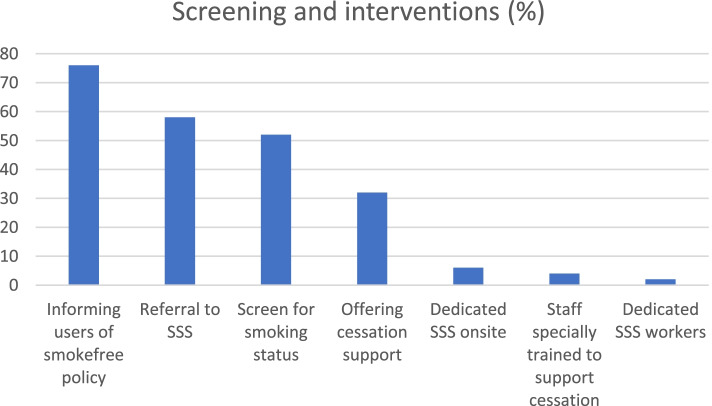
Percentage of service providers (*n* = 99) which screen for smoking and offer different types of support to smokers

All respondents reported having accounted for smoking within some type of policy. Just over half (56%) of services had a standalone smoking policy (i.e., not embedded within another policy); 36% had a health and safety policy that included policies on smoking and 8% had no policy which related to or included smoking. For 58%, the smoking policy was part of broader organisational policy which covered all services as part of that organisation, for the rest it was specific to the individual centre. In response to ‘smokefree’ and environmental policies, nearly all (96%) of respondents reported that in areas where they do not allow smoking, they also include vaping within this restriction; 4% of centres allowed vaping indoors and 1 centre had no policy on vaping. The majority (76%) reported that they inform service users of the smoke free policy (see Fig. 1).

Smoking was permitted in some designated areas by 84% of services, including private indoor spaces such as bedrooms or bathrooms (41%), or public outdoor spaces such as front of premises or outdoor courtyards (61%). 56% reported displaying ‘no smoking allowed’ signs and had no signs about vaping, 32% did not have any signage, 1 service had no signs on smoking restrictions but did display ‘vaping allowed’ signs. The majority (82%) reported that no exceptions were made on where service users could smoke.

Respondents at 62% of services reported that on an average day, staff smoked with service users; 21% reported that this occurred daily, and 38% indicated that this did not happen. Open question responses suggested that staff and service users used the same designated smoking areas, often at the same time. While some centres had rules on not giving service users cigarettes, others said staff would share their own cigarettes with service users. Although some services highlighted that smoking with service users was not encouraged, it was described as acceptable and useful in some situations, e.g., helping to build rapport, to de-escalate a challenging situation, or aid relaxation for a distressed service user. A minority (3%) of centres reported purchasing cigarettes for service users in exceptional circumstances; one centre identified the COVID-19 pandemic as the reason for purchasing cigarettes for people, as a means of encouraging isolation during lockdown.

Staff at 78% of centres reported that the most common problem caused by smoking onsite was cigarette litter; complaints from neighbours about litter was reported by 18% of respondents; disagreements with services users about smoking were reported by 50%, and disagreements between staff about smoking were reported by 22%.

### Screening and recording of smoking status

Figure 1 shows 52% of services screen and record smoking status. Responses to the open-ended question about this highlighted areas of good practice including, rather than simply screening for smoking, using this information proactively by following up on smoking status and asking service users if they would like to quit, and signposting or referring to relevant services (Table 2). Sometimes, this was further followed up by staff at a review or keywork session. Two centres also highlighted that smoking was discussed with service users in the context of barriers to financial management or budgeting for housing.

### Cessation training and resources available to staff

Although some centres described making staff aware of how to refer or signpost service users to cessation services (see Table 2), the majority of centres did not provide specific training to staff on smoking or smoking cessation. As shown in Fig. 1, only 4% of services had specifically trained staff to support cessation. One centre highlighted that training would be needed to enable staff to support smoking cessation among service users, another explained that their training priorities lay with illicit–substance use. No centres reported mandated smoking cessation training as part of their core training.

### Cessation support for service users – links to services

**Fig. 2:**
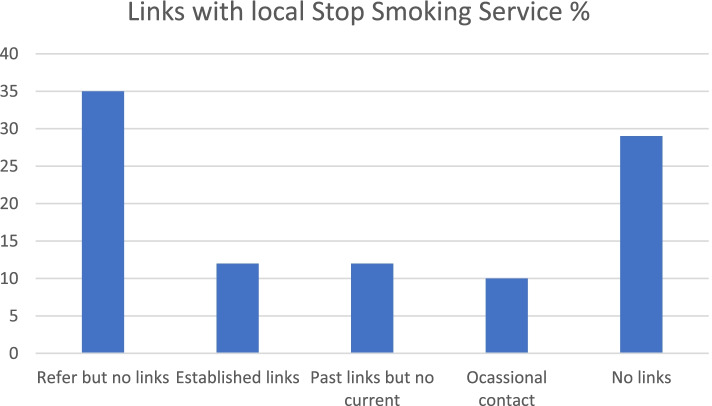
Percentage of service provider level services (*n* = 99) and types of connections to local SSS

Figure 2 shows connections with the local SSS; 35% had no established links but reported that they still signposted people to support, 29% reported having no links at all, 12% reported established links, 12% had past but no current links and 10% reported some occasional contact, 1 centre had connections with their local vape shop.

### Good practice and areas for improvement

In an open response question, survey participants were asked to share what they considered to be examples of innovation and good practice around supporting smoking reduction/cessation. A small number of centres described building a good relationship with a local SSS and highlighting the benefit of doing so (see Table 2). Regularity of access to discussions about smoking and support was a key feature, although as one centre highlighted, it could take time to build relationships. Observing other service users or staff having positive experiences with local services helped with motivation to quit and engagement with services.

It was also identified from open question responses that some centres proactively encouraged service users to vape instead of smoke as a method of reducing the harm from combustible tobacco use. These centres gave positive accounts of their experiences, recounting that service users were receptive to vaping, provided support to each other and in some instances, had reduced their smoking (see Table 2). One centre highlighted a successful collaboration with a local vape store. Others noted their service users’ increased interest in engaging with services which offered free e-cigarettes.

Respondents identified areas of improvement in the open question responses, which related to screening and recording smoking status (Table 2). Of those services which reported not screening for smoking when service users entered the service, some explained that smoking would only be discussed if it was causing a problem or raised by the service user. Others explained that although they did have a smoking question on their initial assessment form to be completed on service entry, this was not always asked. This was usually because staff felt it was inappropriate to ask about smoking when service users presented with other priorities or challenges. In other instances, smoking was asked only as part of a risk assessment, i.e. to gauge fire risk within a residential unit.

## Discussion

This study presents the first overview of how smoking is managed within a sample of UK homeless support services. Respondents identified examples of what they perceived to be good practice and some areas requiring improvement. Survey responses in relation to good practice, i.e., demonstrating that smoking is addressed, considered and reportedly managed, highlight that the majority of services address smoking within some type of policy, be it environmental policies (e.g., smoking restrictions) or addressing smoking amongst service users (e.g. offering support). These were either individual policies or as part of a broader policy. The majority reported that they informed services users of the smoking policies. Half of the services reported that they identify and record the smoking status of service users, with around a third proactively signposting service users for specialist stop smoking support and others waiting for service users to request support. A minority also proactively encouraged service users to vape instead of smoke and some respondents viewed this as helpful to service users.

Some had established links with SSS and a third referred on but had no links. Even without established links, this is good practice, and at the current time in the UK the SSS are the recommended service for helping people to quit smoking. Some also reported setting smoking cessation support within broader health workshops or events (e.g., for tuberculous). Embedding cessation advice in other activities and events may be advantageous in this setting because our findings show that, although screening and making referrals was part of the policy for some, open responses indicate that this could sometimes go unchecked if service users were distressed or presented with other complex issues at the time of screening.

Responses also indicate areas for improvement, i.e., areas which could be improved to better facilitate smoking reduction and or quitting. A large percentage did not distinguish smoking from vaping (4% did distinguish vaping from smoking in restricted smoking areas), and no services had a separate vaping policy. Smoking was allowed in most of the services in designated private areas and also communal outdoor areas. Staff identified cigarette litter as a problem, and smoking also prompting complaints from neighbours and creating disharmony between staff and service users. Staff did report smoking with service users and there was some indication in the open responses of staff purchasing tobacco during the COVID-19 pandemic. A minority had established links with SSS and where service users had been referred but there were no clear measurable effects of benefit, and nobody reported that they evaluated the impact of these links and referrals.

Overall, our results corroborate those from other studies which have interviewed staff and service users of homeless centres, that smoking cessation is not consistently and comprehensively addressed in homeless services and staff training is not well developed [10–12]. However, it is important to highlight that smoking is considered across various policies. If properly enforced, smoking policies can help to provide environmental protection (e.g., reducing fire risk, protecting bystanders) and also help to reduce the social and culture aspects of smoking which are reported elsewhere as barriers to successful cessation [11, 24, 25]. However, any efforts to reduce smoking in the environment could be undermined by poor practice, such as staff smoking with service users, including sharing cigarettes tacit approval of smoking in restricted areas, and purchasing tobacco on behalf of services users.

Open question responses highlighted, for some, vaping was perceived as an alternative to smoking. There was some indication in the open question responses that vaping had been successful for some service users and, as has been cited elsewhere, preferred over NRT [11, 14]. A living systematic review by Hartmann-Boyce et al. [26] reports that e-cigarettes are an effective smoking cessation aid and are more effective than NRT, but evidence for their use for people with extensive health and social needs is still lacking [12].

The findings presented here contribute to an important evidence gap, as a systematic review has shown that to date, the majority of the evidence derives from the United States [5]. For UK researchers and those working in the sector who are interested in supporting people experiencing homelessness to quit smoking, we provide an insight into what is currently being offered, areas where improvements can be made, and highlight areas of good practice which may be important to maximise for better effect.

However, there are some limitations of our research. First, we invited a large sample of services to take part and received a high percentage of rejections. Therefore, this study may have appealed to services with a particular interest in smoking and its relevance in their service. Second, our study was conducted during the COVID-19 pandemic, this meant that many homeless services were extremely busy and unable to take part and this may have biased the types of services that were able to take the time to be interviewed. The cross-sectional nature of the survey also means we do not capture changes in practice, indeed, several organisations across the health and social sector in England have increased their capacity to offer smoking cessation support to disadvantaged groups because of the hypothesised risk of COVID-19 to people who smoke [27].

## Conclusion

Smoking is considered by many homeless services and several environmental and health policies exist which can prevent and reduce smoking. For smoking to be further reduced within this population, more comprehensive policies and interventions are needed which support the reduction of smoking in these organisations.

## Supplementary Information

**Additional file 1.**

**Additional file 2.**

## Data Availability

The anonymised data will be made available on the London South Bank open access repository upon publication of the manuscript https://openresearch.lsbu.ac.uk.

## References

[1] Jha P, Peto R, Zatonski W, Boreham J, Jarvis MJ, Lopez AD (2006). Social inequalities in male mortality, and in male mortality from smoking: indirect estimation from national death rates in England and Wales, Poland, and North America. Lancet.

[2] Hiscock R, Bauld L, Amos A, Fidler JA, Munafò M (2012). Socioeconomic status and smoking: a review. Ann N Y Acad Sci.

[3] Di Cesare M, Khang Y-H, Asaria P, Blakely T, Cowan MJ, Farzadfar F (2013). Inequalities in non-communicable diseases and effective responses. Lancet.

[4] Marmot M (2020). Health equity in England: the Marmot review 10 years on. BMJ.

[5] Soar K, Dawkins L, Robson D, Cox S (2020). Smoking amongst adults experiencing homelessness: a systematic review of prevalence rates, interventions and the barriers and facilitators to quitting and staying quit. J Smok Cessat.

[6] Adult smoking habits in the UK: 2019. Office for National Statistics; 2020. Available from: https://www.ons.gov.uk/peoplepopulationandcommunity/healthandsocialcare/healthandlifeexpectancies/bulletins/adultsmokinghabitsingreatbritain/2019#strengths-and-limitations. Cited 2020 Sep 14.

[7] Lewer D, Aldridge RW, Menezes D, Sawyer C, Zaninotto P, Dedicoat M (2019). Health-related quality of life and prevalence of six chronic diseases in homeless and housed people: a cross-sectional study in London and Birmingham, England. BMJ Open.

[8] Tucker JS, Shadel WG, Golinelli D, Mullins L, Ewing B (2015). Sniping and other high-risk smoking practices among homeless youth. Drug Alcohol Depend.

[9] White JM, Irvine RJ (1999). Mechanisms of fatal opioid overdose. Addiction.

[10] Vijayaraghavan M, Elser H, Frazer K, Lindson N, Apollonio D. Interventions to reduce tobacco use in people experiencing homelessness. Cochrane Tobacco Addiction Group, editor. Cochrane Database Syst Rev. 2020; Available from: http://doi.wiley.com/10.1002/14651858.CD013413.pub2. Cited 2020 Dec 15. 10.1002/14651858.CD013413.pub2 PMC8130995 33284989

[11] Collins SE, Orfaly VE, Wu T, Chang S, Hardy RV, Nash A (2018). Content analysis of homeless smokers’ perspectives on established and alternative smoking interventions. Int J Drug Policy.

[12] Cox S, Ford A, Li J, Best C, Tyler A, Robson DJ (2021). Exploring the uptake and use of electronic cigarettes provided to smokers accessing homeless centres: a four-centre cluster feasibility trial. Public Health Res.

[13] Maddox S, Segan C (2017). Underestimation of homeless clients’ interest in quitting smoking: a case for routine tobacco assessment. Health Promot J Austr.

[14] Dawkins L, Ford A, Bauld L, Balaban S, Tyler A, Cox S (2019). A cross sectional survey of smoking characteristics and quitting behaviour from a sample of homeless adults in Great Britain. Addict Behav.

[15] Hartmann-Boyce J, Chepkin SC, Ye W, Bullen C, Lancaster T. Nicotine replacement therapy versus control for smoking cessation. Cochrane Tobacco Addiction Group, editor. Cochrane Database Syst Rev. 2018;2019(1) Available from: http://doi.wiley.com/10.1002/14651858.CD000146.pub5. Cited 2022 Apr 8. 10.1002/14651858.CD000146.pub5 PMC6353172 29852054

[16] Hartmann-Boyce J, McRobbie H, Butler AR, Lindson N, Bullen C, Begh R, et al. Electronic cigarettes for smoking cessation. Cochrane Tobacco Addiction Group, editor. Cochrane Database Syst Rev. 2021;2022(4) Available from: http://doi.wiley.com/10.1002/14651858.CD010216.pub6. Cited 2022 Apr 8. 10.1002/14651858.CD010216.pub5 PMC8092424 33913154

[17] Hartmann-Boyce J, Livingstone-Banks J, Ordóñez-Mena JM, Fanshawe TR, Lindson N, Freeman SC, et al. Behavioural interventions for smoking cessation: an overview and network meta-analysis. Cochrane Tobacco Addiction Group, editor. Cochrane Database Syst Rev. 2021; Available from: http://doi.wiley.com/10.1002/14651858.CD013229.pub2. Cited 2022 Apr 8. 10.1002/14651858.CD013229.pub2 PMC11354481 33411338

[18] McNeill A, Brose L, Calder R, Bauld L, Robson D (2019). Vaping in England: an evidence update, February 2019. A report commissioned by Public Health England.

[19] Smoking and health 2021: a coming of age for tobacco control?. Royal College of Physicans; 2021. Available from: https://www.rcplondon.ac.uk/projects/outputs/smoking-and-health-2021-coming-age-tobacco-control. Cited 2022 Apr 1.

[20] Tobacco: preventing uptake, promoting quitting and treating dependence. National Institute for Health and Care Excellence; 2021. Available from: https://www.nice.org.uk/guidance/ng209. Cited 2021 Dec 1. 36745727

[21] Dawkins L, Bauld L, Ford A, Robson D, Hajek P, Parrott S (2020). A cluster feasibility trial to explore the uptake and use of e-cigarettes versus usual care offered to smokers attending homeless centres in Great Britain. Leroyer C, editor. PLoS One.

[22] Blackwell AKM, Kosīte D, Marteau TM, Munafò MR (2020). Policies for tobacco and E-cigarette use: a survey of all higher education institutions and NHS trusts in England. Nicotine Tob Res.

[23] Brief guide: smokefree policies in mental health inpatient services. Care Quality Commission; 2018. Available from: https://www.cqc.org.uk/sites/default/files/20170109_briefguide-smokefree.pdf. Accessed 20 Apr 2021.

[24] Okuyemi K, Caldwell A, Thomas J, Born W, Richter K, Nollen N (2006). Homelessness and smoking cessation: insights from focus groups. Nicotine Tob Res.

[25] Reitzel LR, Kendzor DE, Nguyen N, Regan SD, Okuyemi KS, Castro Y (2014). Shelter proximity and affect among homeless smokers making a quit attempt. Am J Health Behav.

[26] Hartmann-Boyce J, McRobbie H, Lindson N, Bullen C, Begh R, Theodoulou A, et al. Electronic cigarettes for smoking cessation. Cochrane Tobacco Addiction Group, editor. Cochrane Database Syst Rev. 2020; Available from: http://doi.wiley.com/10.1002/14651858.CD010216.pub4. Cited 2020 Dec 15. 10.1002/14651858.CD010216.pub4 PMC8094228 33052602

[27] Cox S, Ward E, Ross L, Notley C (2021). How a sample of English stop smoking services and vape shops adapted during the early COVID-19 pandemic: a mixed-methods cross-sectional survey. Harm Reduct J.

