# Supplementary material for: A cross-sectional survey of smoking and cessation support policies in a sample of homeless services in the United Kingdom
Source: BMC Health Serv Res. 2022 May 13;22:635. doi: 10.1186/s12913-022-08038-7 (PMC9098377; doi:10.1186/s12913-022-08038-7)
Supplement: Supplementary file 1 — Additional file 1. [file 12913_2022_8038_MOESM1_ESM.docx]

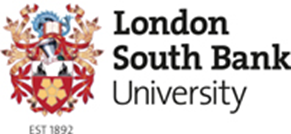
**
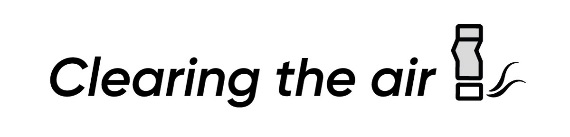
**

**Please complete this survey if you have an overview of how smoking is treated within your centre or site.**

**THE CENTRE**

| **Centre details** |
| --- |
| Town: |
| Post code (required to allow analysis of regional patterns): |
| Number of staff employed at the current time including volunteers: |
| Number of service users visiting your service on an average day: |

| **What type of provision do you offer (tick all that apply)?** | | |
| --- | --- | --- |
| Day centres (food, showers, daily key-work, signposting to other services) | Assessment centres | Mental health support  Including counselling therapies |
| Emergency night shelters (e.g. ‘SWEP beds’) | Housing First schemes | Physical health support |
| Supported housing (e.g. hostels) | Winter shelter | Employment support and advice |
| Crash pads | Accommodation services  Including tenancy sustainment support | Substance use treatment services |
| Temporary housing | Employment services |  |
| **Other:** | | |
|  | | |

**THE FOLLOWING QUESTIONS ARE BASED ON YOUR CENTRE’S POLICY**

| **Do you have an organisational-wide smoking policy or vaping policy? This may be part of another policy (e.g., health and safety, fire safety) or a standalone policy?** | |
| --- | --- |
| Yes | No |

| **If smoking covered by another policy, which policy is that? (tick all that apply)** | |
| --- | --- |
| Health and safety |  |
| Physical health |  |
| Health and wellbeing |  |
| Fire hazard and risk |  |
| Other (name): |  |

| **(For those with a smoking policy) How long has it been in place and when was it last updated? Would you be interested in working with us to develop this policy?** |
| --- |
|  |
|  |

| **For those with a smoking policy) Is this policy specific to this centre/site or does it fall under a broader organisational policy?** | |
| --- | --- |
| Specific to this centre or site | Part of a broader organisational policy |

| **If you do not have a smoking policy do you wish to develop one? Would you be interested in hearing from our team about this? (Take name of contact)** |
| --- |
|  |
|  |

| **If you have a written smoking policy, where is it available?** |
| --- |
| **To staff:** |
|  |
| **To service users:** |
|  |
| **To the public:** |
|  |

| **Is your smoking policy publicly available? If so how?** |
| --- |
|  |
|  |

| **Are rules the same for smoking and vaping (e.g., e-cigarettes are included under a broad definition of smoking)?** |
| --- |
|  |
|  |
|  |
|  |

| **If the rules on vaping differ, please tell us how:** |
| --- |
|  |
|  |
|  |

| **Is there any variation on the rules dependent on unpredictable events, for example bad weather, service user distress? Please describe** |
| --- |
|  |
|  |
|  |
|  |
|  |

| **As far as you are aware, has there been any evaluation on the impact of smoking? (e.g., cigarette litter, room discolouration, offering stop smoking support). If so please tell us what happened, when and how this worked.** |
| --- |
|  |
|  |
|  |
|  |
|  |

**THE FOLLOWING QUESTIONS ARE BASED ON THE CENTRE’S PRACTICE**

| **Is there a standard requirement that your staff/volunteers should ask services users about their smoking?** |
| --- |
|  |
|  |
|  |

| **Does your centre’s smoking policy or *practice* include the following? (tick all that apply)** |
| --- |
| Informing patients about the smoke free policy upon use of the centre |
| Identifying and recording smoking status on admission |
| Offering all smokers support to quit or cut down |
| Referral of smokers to community stop smoking services |
| Prohibiting smoking in designated places |
| Permitting smoking only in certain areas |
| Not applicable |
|  |
| **How are transgressions to the smoking policies treated?** |
|  |
|  |

| **Is stop smoking advice offered to service users as standard practice? (If so, what does this involve)** |
| --- |
| Frontline staff who are trained to support people through a quit attempt or period of abstinence |
| Dedicated smoking cessation workers |
| A dedicated smoking cessation service/clinic |
| None of the above |
| Don't know |
| Other: |
|  |
|  |
|  |

| **What training (if any) is provided to staff and volunteers on smoking and smoking cessation and how this is monitored?** |
| --- |
|  |
|  |
|  |

| **What proportion of the centre’s frontline staff are trained in providing stop smoking support?** |
| --- |
| All of them |
| A majority of them |
| Around half of them |
| A minority of them |
| None of them |
| Don't know |

| **Is there a rule or line of advice for staff around smoking with or in front of service users? (e.g., do you have a blanket rule that says staff should not smoke in front of service users)** |
| --- |
|  |
|  |
|  |
|  |

| **9. Are the rules about where staff can smoke different from the rules for service users?** |
| --- |
|  |
|  |

**THE FOLLOWING QUESTIONS ARE ABOUT THE CENTRE’S ENVIRONMENT**

| **Where are those using your service currently permitted to smoke? (tick all that apply)** |
| --- |
| Outside – front/back of the building |
| In gardens/courtyards (where applicable) |
| Outside but only in designated smoking shelters |
| In a smoking room/designated area (inside)(please describe) |
| Bedroom or secure night accommodation space only |
| Other (please describe) |
| Nowhere on the premises |
| Don't know |
| **Description/comments** |
|  |
|  |
|  |
|  |

| **Thinking about what happens in practice on an average day in your centre how often are services users found:** | | | | | | |
| --- | --- | --- | --- | --- | --- | --- |
|  | At least once a day | At least once a week | At least once a month | Less often | Never | Don't know |
| Smoking in bedrooms or bathrooms |  |  |  |  |  |  |
| Smoking in gardens or courtyards |  |  |  |  |  |  |
| Smoking in indoor communal areas (other than bedrooms) |  |  |  |  |  |  |
| Smoking in somewhere else smoking is banned |  |  |  |  |  |  |
| Staff smoke with residents/service users |  |  |  |  |  |  |

| **From your observations what percentage or number of staff at your centre also smoke?** | |
| --- | --- |
| % | Unsure |

| **From your observations what percentage or number of your staff vape (use an e cigarette)?** | |
| --- | --- |
| **%** | Unsure |

| **Is it a requirement for smoking/vaping signs to be up in buildings?** |
| --- |
| Yes (’No smoking’ signs only, no signs about vaping) |
| Yes (Both ‘no smoking’ & ‘no vaping’ signs) |
| Yes (‘No smoking’ but ‘vaping allowed’ signs) |
| No |

| **How would you describe your centre’s contact with the local stop smoking services?** |
| --- |
| We have well established links with our stop smoking services |
| We have had some links with them in the past |
| We occasionally work with them |
| We refer people to the service but do not have a formal link |
| We do not have any links with the stop smoking services |
| Do not know |
| **Description/comment:** |
|  |
|  |

| **Do you have any links with your local vape store? (If yes provide details)** |
| --- |
|  |
|  |

| **In the past, have you experienced any of the following problems at your centre (say all that apply)** |
| --- |
| Cigarette littering |
| Complaints from neighbours about smoking or littering |
| Disagreements/conflicts with service users about smoking |
| Disagreements/conflicts with staff around smoking |
| None of the above |
| **Description/comments** |
|  |

**THINKING AHEAD**

| **We have developed some smoking and cessation information videos for third sectors organisations, would you be interested in receiving a link to these and details of our other projects?** |
| --- |
| **Yes/No** |

| **We would like to share examples of innovation and good practice around supporting smoking within homeless centres. If you have any examples that you think others would value, please share**: |
| --- |
|  |
|  |
|  |
|  |
|  |

**Thank you for taking the time to complete this survey. The aggregated findings of the survey will be shared nationally and regionally, and every trust will receive its own confidential tailored feedback.**
