# Supplementary material for: A cross-sectional survey of smoking and cessation support policies in a sample of homeless services in the United Kingdom
Source: BMC Health Serv Res. 2022 May 13;22:635. doi: 10.1186/s12913-022-08038-7 (PMC9098377; doi:10.1186/s12913-022-08038-7)
Supplement: Supplementary file 2 — Additional file 2. [file 12913_2022_8038_MOESM2_ESM.docx]

**Supplementary material**

**Supported housing** describes temporary or medium-term communal or clustered housing with staff support provided. Some degree of shared space is common, but accommodation may be entirely self-contained.

**Day centres** are building-based, non-residential, often ‘drop-in’ services that provide advice and support with housing and related issues alongside facilities such as food, showers, laundry, internet and communal social space.

**Emergency night shelters** offer short term shelter and a bed, often in a shared space, for people who would otherwise sleep rough.

**Crash pad** is the least formalised descriptor but is commonly used to describe an emergency bed that can be accessed by someone who would otherwise sleep rough, often within another service type. Occasionally, this phrase is particularly used for a bed reserved for young people at risk.

**Employment support** describes activities undertaken to help prepare someone to (re)enter work and may comprise IT training, CV-writing support and practical skills training.

**Mental health support** within a homelessness service may be delivered by peripatetic external staff or provided in-house. Services may also undertake activities designed to support mental wellness.

**Housing/accommodation support** comprises advice on accessing housing and, in some cases, supported advocacy, such as accompanying someone to appointments (and may include support with claiming benefits).

**Physical health support** within a homelessness service may be delivered by peripatetic external staff or provided in-house. In some cases, specialist screening and treatment services may attend generalist services to encourage uptake, such as mobile screening services. Services may also undertake activities designed to support physical wellness.

**Substance use** (drug and alcohol) support may comprise supported access to treatment, provision of mutual aid support groups and harm minimisation work.

**Street outreach** is work to engage with people in the areas where they sleep rough, primarily to check their wellbeing and to facilitate access to shelter or accommodation.

**Winter shelters** are emergency night shelters that open only during colder months. In many areas, there will be a network of winter shelters opening on alternating nights.
